# Visualizing Peripheral Nerve Regeneration by Whole Mount Staining

**DOI:** 10.1371/journal.pone.0119168

**Published:** 2015-03-04

**Authors:** Xin-peng Dun, David B. Parkinson

**Affiliations:** 1 Plymouth University Peninsula Schools of Medicine and Dentistry, Plymouth, Devon, United Kingdom; 2 Hubei University of Science and Technology, Xian-Ning City, Hubei, China; Hertie Institute for Clinical Brain Research, University of Tuebingen., GERMANY

## Abstract

Peripheral nerve trauma triggers a well characterised sequence of events both proximal and distal to the site of injury. Axons distal to the injury degenerate, Schwann cells convert to a repair supportive phenotype and macrophages enter the nerve to clear myelin and axonal debris. Following these events, axons must regrow through the distal part of the nerve, re-innervate and finally are re-myelinated by Schwann cells. For nerve crush injuries (axonotmesis), in which the integrity of the nerve is maintained, repair may be relatively effective whereas for nerve transection (neurotmesis) repair will likely be very poor as few axons may be able to cross between the two parts of the severed nerve, across the newly generated nerve bridge, to enter the distal stump and regenerate. Analysing axon growth and the cell-cell interactions that occur following both nerve crush and cut injuries has largely been carried out by staining sections of nerve tissue, but this has the obvious disadvantage that it is not possible to follow the paths of regenerating axons in three dimensions within the nerve trunk or nerve bridge. To try and solve this problem, we describe the development and use of a novel whole mount staining protocol that allows the analysis of axonal regeneration, Schwann cell-axon interaction and re-vascularisation of the repairing nerve following nerve cut and crush injuries.

## Introduction

Sciatic nerve crush (axonotmesis) or transection (neurotmesis) injury paradigms have been widely used as models to study the mechanisms controlling peripheral nerve regeneration and repair in both rats and transgenic mice, and these experimental approaches have identified both axonal and Schwann cell-dependent mechanisms for controlling nerve regeneration. The accurate measurement of axonal re-growth and interactions between axons and Schwann cells is, however, difficult using the standard approach of staining cryosections of the injured nerve; serial sections must be stained and it may be difficult both to identify the injury site and, more importantly, to follow the path of individual axons that may move in or out of the section plane.

Peripheral nerve trauma induces a fairly well characterised series of events in the nerve distal to the site of injury; axons degenerate, Schwann cells convert to a repair supportive phenotype, proliferate and begin the process of myelin breakdown and phagocytosis that is taken to completion by the influx of macrophages [1–4]. For crush injuries, where the structure of the nerve is maintained, axonal regeneration is largely successful and function may be fully restored in rodents 3–4 weeks after injury [5–7]. For nerve transection injuries, the situation is different as the two ends of the cut nerve separate and a nerve bridge, comprising mostly of Schwann cells and nerve fibroblasts, forms to allow axons to cross from the proximal to the distal stump [8]. Unsurprisingly, functional repair is much poorer in nerve transection injuries even with the surgical apposition of the nerve ends following trauma [9, 10]. As described above, analysing the mechanics of axonal growth and Schwann cell-axon interactions is not easy using conventional staining of thin cryostat or paraffin sections, axons move in and out of plane, and the nerve or nerve bridge cannot be visualised as a whole. The technique of whole mount *in situ* hybridisation has been widely used for the study of gene expression during embryogenesis in the mouse, chicken, fruit fly and zebrafish and has been successfully modified to use antibody staining for the investigation of axon path-finding during embryonic development, known as whole mount staining (or immunohistochemistry) [11].

In this paper, we describe the use of a novel whole mount staining protocol that is suitable for the study of the events following nerve crush and cut injuries. We demonstrate its use in analysing the early events of peripheral nerve regeneration, namely axon regrowth, Schwann cell-axon interaction in both the proximal stump and nerve bridge, and the angiogenesis within the nerve following injury.

## Materials and Methods

### Animal husbandry and peripheral nerve surgery

All work involving animals was carried out according to Home Office regulation under the UK Animals (Scientific Procedures) Act 1986. Ethical approval for all experiments was granted by Plymouth University Animal Welfare and Ethical Review Board and all efforts were made to minimise animal suffering. C57BL/6 mice were purchased from Charles River UK limited. Mice were anaesthetised with isoflurane, the right sciatic nerve exposed and for the nerve cut procedure transected at approximately 0.5 cm distal to the sciatic notch. No re-anastamosis of the severed nerve, either by suture or by fibrin glue, was performed in the nerve transection procedures in these experiments. This allowed analysis of axons crossing the nerve bridge that forms between the retracted proximal and distal nerve stumps [8, 12]. For nerve crush experiments, the sciatic nerve was crushed once for 30 seconds using a pair of delicate forceps (Fine Science Instruments; 0.4mm tip angled, Cat no. 11063-07), and again for 30 seconds at the same site but orthogonal to the initial crush. All animals undergoing surgery were given appropriate post-operative analgesia and monitored daily. At the indicated timepoint post-surgery for each experiment described, animals were euthanased humanely by cervical dislocation in accordance with UK Home Office regulations.

### Whole mount staining

At the described timepoints following surgery, nerves were removed and fixed in 4% paraformaldehyde for 5 hours at 4°C. Following fixation, nerves were then washed in PTX (1% Triton X-100 (Sigma, T9284) in phosphate buffered saline (PBS)) three times for 10 minutes each time. To ensure better antibody penetration for nerve crush samples, the epineurium was removed in these preparations after washing in PTX. Nerves were subsequently incubated with blocking solution (10% foetal bovine serum (FBS) in PTX) overnight at 4°C. The following day, nerves were transferred into primary antibodies in PTX containing 10% FBS and incubated for 48h-72h at 4°C with gentle rocking. Primary antibodies used for the experiments are neurofilament heavy chain (1:1000, Abcam, ab4680), S100β (1:100, DAKO, Z0311), Ki67 (1:100, Abcam, ab15580) and myelin basic protein (1:100, Santa Cruz Biotechnology, sc-13912). After the incubation, nerves were washed with PTX three times for 15 minutes each wash, followed by washing in PTX for 6 hours at room temperature, with a change of PTX every 1 hour. Secondary antibodies (1:500, Invitrogen) and Hoechst dye (1:1000, Invitrogen) were diluted in PTX containing 10% FBS, and incubated with the nerves for 48h at 4°C with rocking. Next, nerves were washed in PTX three times for 15 minutes each, followed by washing in PTX for 6 hours at room temperature, changing the PTX each hour, and then washed overnight without changing PTX at 4°C. For FluoroMyelin Red (Invitrogen, F34652) staining, FluoroMyelin Red in PBS (1:300) was used to stain the nerves for 30 minutes at room temperature. When staining was complete, the nerve was washed 3 times for 10 minutes each in PBS. Nerves were cleared sequentially with 25%, 50%, 75% glycerol (Sigma, G6279) in PBS between 12–24h for each glycerol concentration. Following clearing, nerves were mounted in CitiFluor (Agar Scientific, R1320) for confocal imaging.

### Imaging

Images were obtained with a Zeiss LSM510 confocal microscope. Several Z-series were captured, covering the entire field of interest. The individual series were then flattened into a single image for each location and combined into one image using Adobe Photoshop software (Adobe Systems). Axonal growth were measured using Nikon software (NIS-elements software and Nikon Eclipse 80i fluorescent microscope) after the images were taken.

## Results

### Measuring the speed of axonal regrowth in crush or transection injured mouse sciatic nerve using whole mount staining

**Fig 1:**
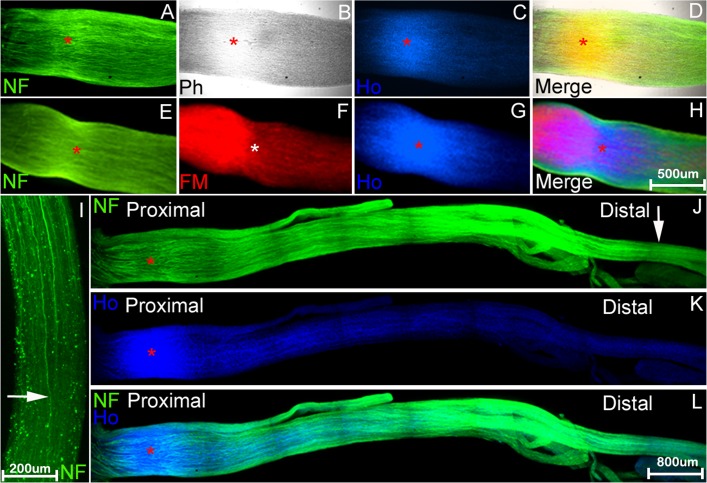
Identifying the nerve injury site and leading regrowing axons after nerve crush injury using whole mount staining. Whole mount stained mouse sciatic nerve preparations at 5 days after crush injury. A-H: visualisation of crush site (indicated by red or white asterisk) by staining with neurofilament heavy chain (NF) antibody (A and E), Hoechst (Ho) dye (C and G), phase contrast (B) or Fluoromyelin, FM (F). Merged overlay panels in D (A-C) and H (E-G). I-L: neurofilament (I and J) and Hoechst (K) whole mount stain of nerve preparations distal to the crush injury showing the leading regenerating axons, indicated by white arrow, in J, and at higher power in panel I. L: merged overlay of panels J and K. In panels A-H, the proximal side is to the left and distal side to the right. In I, the proximal side is up and distal side to the bottom of the panel.

Nerve crush injury or axonotmesis has been used more frequently for measuring the speed of axonal regrowth and functional recovery as, at least in the rodent system, there is an almost full recovery of motor and sensory function. We have tested the whole mount staining protocol on mouse sciatic nerve samples following crush injury using neurofilament heavy chain antibody, Fluoromyelin red (a commercially available water-soluble fluorescent dye that selectively stains the lipid component of myelin) and counterstaining with Hoechst dye. In initial experiments, neurofilament antibody and Fluoromyelin/Hoechst stain penetration was poor, except at the crush site, even with longer incubation times, and so we removed the epineurium from the nerve after paraformaldehyde fixation and PTX wash. Following this modification, neurofilament and Fluoromyelin/Hoechst staining was much improved. Fig. 1 shows an example of a preparation stained at 5 days following nerve crush injury. The site of the nerve crush injury can be easily distinguished by phase microscopy (crush point marked by asterisk). The crush site may also be identified by Fluoromyelin stain (myelin breakdown at this timepoint distal to the site of injury) and Hoechst stain (proliferation of cells at the crush point). Neurofilament stained regenerating axons can be seen re-growing through the nerve (Fig. 1I and J).

**Fig 2:**
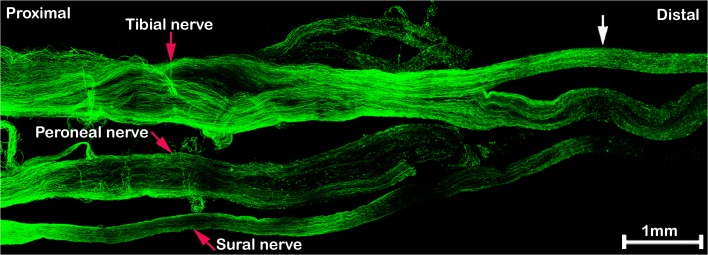
Re-growing axons enter the tibial nerve before other distal branches of the sciatic nerve. The sciatic nerve is stained with neurofilament antibody 5 days after nerve crush injury. At this timepoint, regenerating axons have grown much further within the tibial branch of the distal nerve (indicated by the white arrow) as compared to peroneal and sural branches.

The speed of axonal regrowth can easily be measured after localizing the crush site and the front of axons. We have measured the distance migrated of leading axons in the tibial nerve from the crush site 5 and 7 days after sciatic nerve crush (1.157±0.062cm and 1.893±0.011cm (n = 5), respectively). An interesting finding is that it appears that re-growing axons apparently enter the tibial nerve earlier than other distal branches of the sciatic nerve (Fig. 2).

**Fig 3:**
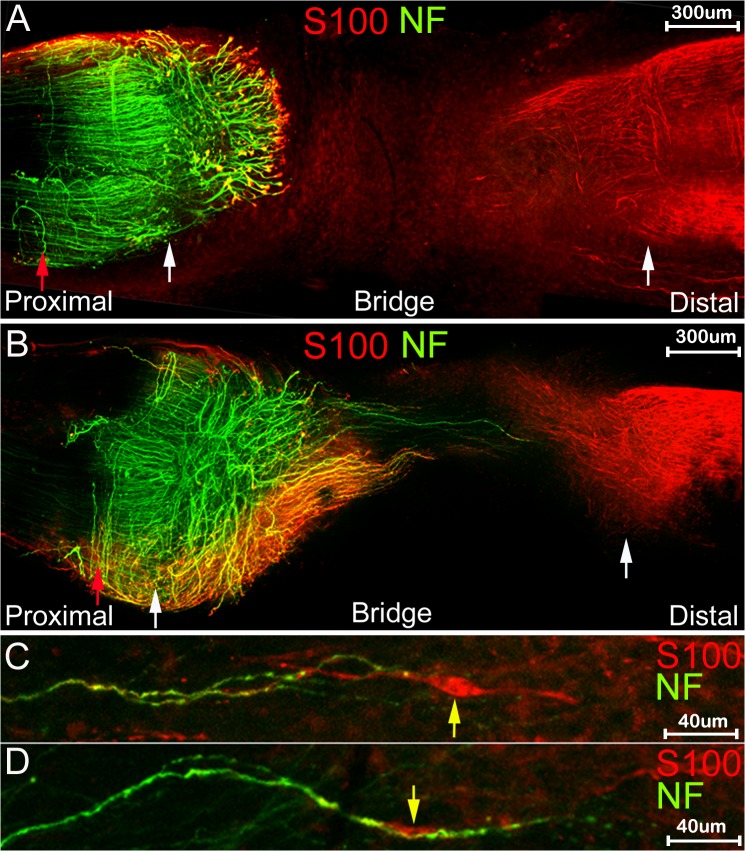
Whole mount staining of transected sciatic nerve at 5 and 7 days after injury. White arrows mark the proximal (left) and distal (right) nerve stumps and the red arrow within the proximal part of the nerve shows the limit of antibody penetration within this part of the nerve. A and B: whole mount stain of nerve preparations using neurofilament (NF) and S100β (S100) antibodies at 5 days (A) and 7 (B) days after nerve transection. C and D: higher magnification pictures of neurofilament and S100β stain showing interaction of distal Schwann cells (indicated by yellow arrow) with axons.

**Fig 4:**
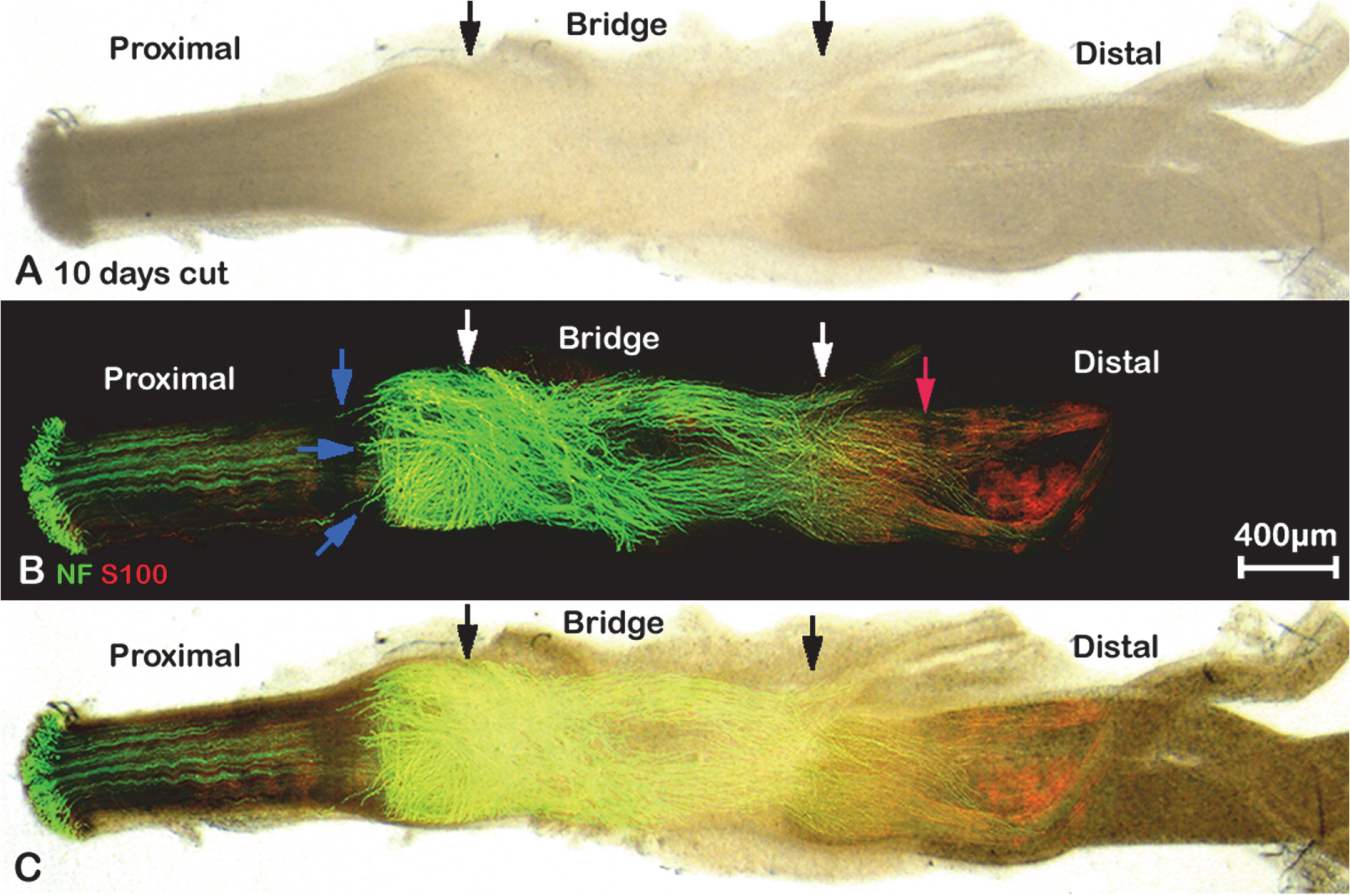
Whole mount staining of transected sciatic nerve preparation after 10 days injury showing misdirected regrowing axons. A: phase image showing the structure of transected sciatic nerve, black arrows mark the proximal (left) and distal (right) nerve stumps. B: neurofilament (NF) and S100β (S100) antibodies whole mount staining shows regenerating axons in the nerve bridge and misdirected regrowing axons in both proximal and distal nerve stumps, blue arrows indicate large axon bundles that have turned 180 degrees and have grown back along the outside of the proximal nerve stump. Axons that have crossed the nerve bridge at this timepoint can be seen entering the distal stump of the nerve (marked by white arrow), but some also can be seen growing outside of the distal part of the nerve. Red arrow shows the distance of neurofilament/S100β antibody penetration within the distal part of the nerve. C: merged image of panels A and B.

**Fig 5:**
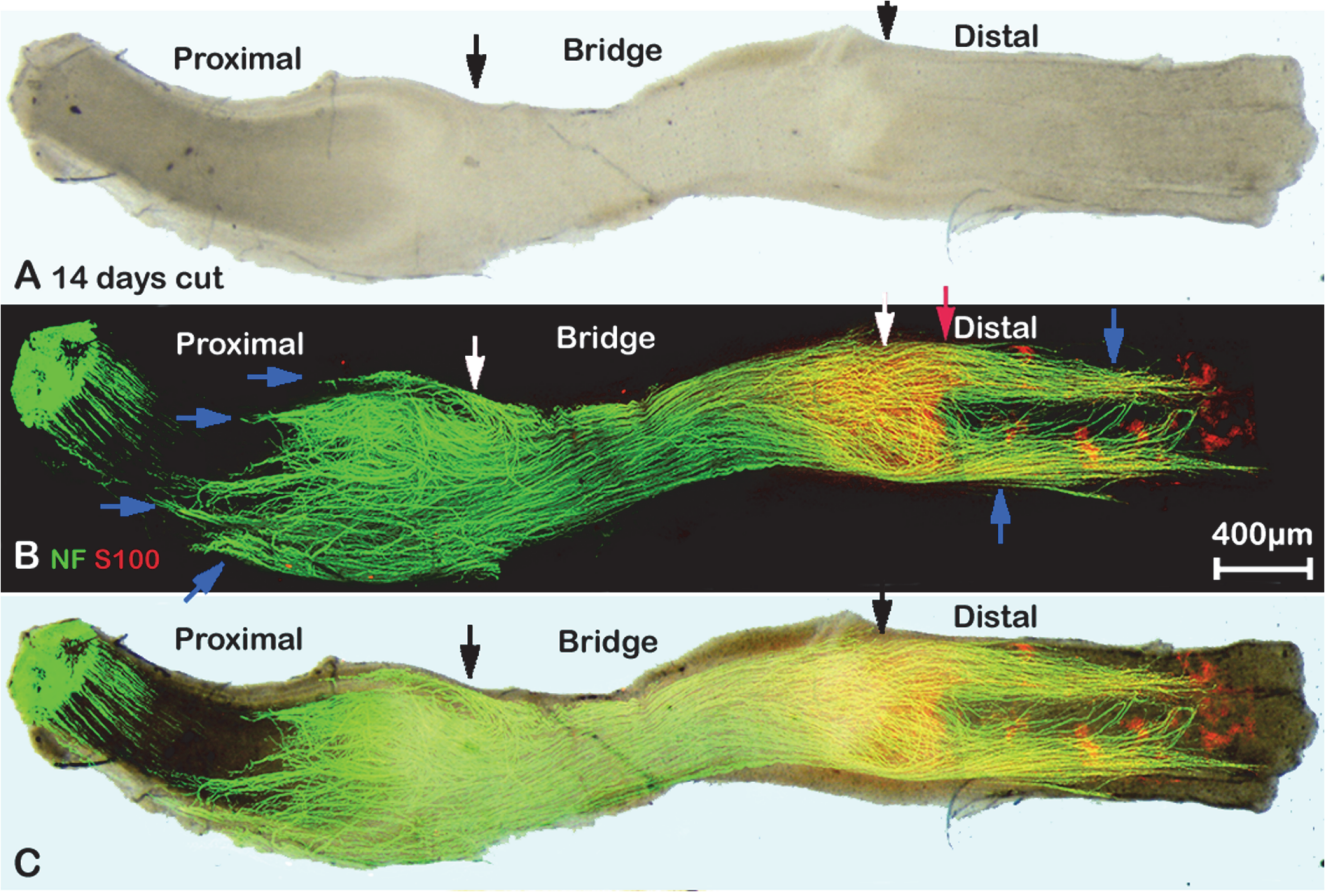
Whole mount staining of transected sciatic nerve preparation after 14 days injury showing misdirected regrowing axons. A: phase image showing the structure of transected sciatic nerve, black arrows mark the proximal (left) and distal (right) nerve stumps. B: neurofilament (NF) and S100β (S100) antibodies whole mount staining show regenerating axons in the nerve bridge and misdirected regrowing axons in both proximal and distal nerve stumps, blue arrows indicate large axon bundles that have turned 180 degrees and have grown back along the outside of the proximal nerve stump. Axons that are growing outside of the distal part of the nerve (blue arrows) can be seen more clearly at 14 days after injury. White arrows mark the proximal (left) and distal (right) nerve stumps. Red arrow shows the distance of neurofilament/S100β antibody penetration within the distal part of the nerve. C: merged image of panels A and B.

Although repair is limited after sciatic nerve transection, this model is still widely used for studying the repair mechanism after severe damage (neurotmesis) of the peripheral nerve. Retraction of the ends of the transected nerve requires the formation of a new nerve bridge between the proximal and distal stumps across which the axons cross to re-enter the distal part of the nerve [8, 12]. We have performed whole mount staining at different timepoints following nerve transection (4, 5, 7, 10, 14 days and 3 months) on transected mouse sciatic nerves. For these experiments, no re-anastamosis of the proximal and distal nerve stumps was performed, in order to view the regeneration of axons across the nerve bridge that forms under such circumstances [8,12]. The presence of an intact epineurium limits antibody penetration into the proximal and distal stumps of the nerve, however removal of the epineurium in the nerve stumps would also remove axons that are growing outside of the nerve that we wish to visualise. Clear staining of axons and Schwann cells is achieved in the nerve bridge by neurofilament heavy chain and S100β antibodies respectively without removing the epineurium, however, only a short distance can be stained at both ends of the proximal and distal stump which still contains the intact epineurium. This distance is shown between the white and red upward arrows for the proximal nerve stump in Fig. 3A and 3B and is the distance between the white and red downward arrows for the distal stump immunofluorescence in Figs. 4B and 5B.

**Fig 6:**
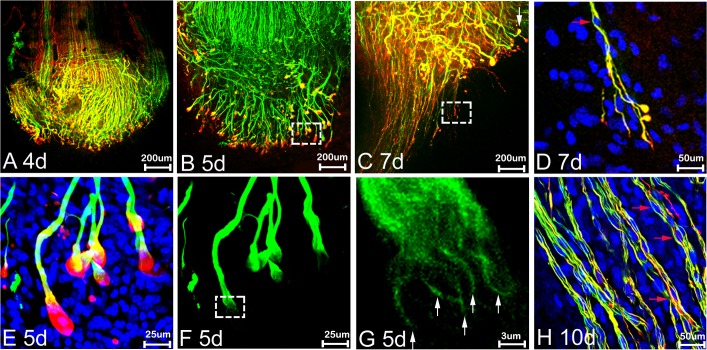
Partnership of Schwann cells and axons during regeneration. Neurofilament (NF; green), S100β (S100; red) and Hoechst (blue) labelling of axons and Schwann cells at times shown after sciatic nerve transection. A-E: the front edges of the regenerating axons are covered by Schwann cell processes at 4, 5 and 7 days (d). At days 4 and 5, Schwann cell processes (red) form a ‘ball-like’ structure at the tip of axon bundles (A, B and E) whereas at 7d (C and D), fine Schwann cell processes appear to proceed in front of the axons. D: higher magnification of boxed area shown in panel C to show Schwann cell leading processes proceeding in front of axons and guiding axons across the nerve bridge. E and F: higher magnification from the boxed area of panel B. G: higher magnification of boxed area in panel F showing the axonal bundles, white arrows in panel G indicate apparent individual axons. Red arrows in D and H show elongated Schwann cell bodies held by axons crossing the nerve bridge. Upon further axon growth in panel H at 10d, elongated Schwann cell bodies can clearly be seen held by axons in the nerve bridge. In all the panels, the proximal side is up and distal to the bottom of the picture.

The initial transection sites from both proximal and distal nerve stumps can be identified using neurofilament heavy chain and S100β antibody staining (Fig. 3A and 3B, indicated by white arrows) at times up to 7 days following transection injury by several criteria. Firstly, the newly formed nerve bridge is much narrower compared to the proximal and distal nerve stumps. Secondly, the alignment of the axons in the proximal stump is always straighter than the regrowing axons, and thirdly, Schwann cells migrating out from the distal stump into the nerve bridge have a typical bipolar shape (Fig. 3C and 3D). In agreement with previous studies using staining of sections [12, 13] that rapid axonal regrowth starts from 4 days after nerve transection (Fig. 6A). We can clearly see the regrowth on day 5 after transection (Fig. 6B). On day 5, leading axons have grown 0.341±0.044 mm into the nerve bridge (n = 5). By day 7, leading axons have grown 1.453±0.194 mm (n = 5). The transection site for the distal stump is still recognizable 10 or 14 days after nerve cut by neurofilament heavy chain antibody staining (Figs. 4B and 5B) and the cut site in the proximal stump can be easily recognized in phase images (Figs. 4A and 5A).

### Misdirection of re-growing axons in transected sciatic nerve

Using whole mount staining, the pattern of axonal re-growth can be clearly revealed at different time points following injury. We have performed whole mount staining at the time points of 4, 5, 7, 10, 14 days and 3 months after sciatic nerve transection. At 5 days, axons appear to be growing bundled together (Figs. 3A, 6B, 6E and 6F). Axons forming large bundles have extended into the nerve bridge at 5 days, and grown a significant distance from the initial cut site of the proximal stump. These axons were clearly covered by Schwann cell processes forming a ball shape at the tip of the axons (Figs. 3A and 6E). By day 7, some axons crossing the bridge appear to be migrating singly and leading axons have almost reached the distal stump of the nerve. The large bundles of axons with ball shaped Schwann cell processes at their tips observed on day 5 appear at 7d to have turned at various angles to the bridge, and many bundles are seen a perpendicular angle to the nerve bridge (Figs. 3B and 6C).

**Fig 7:**
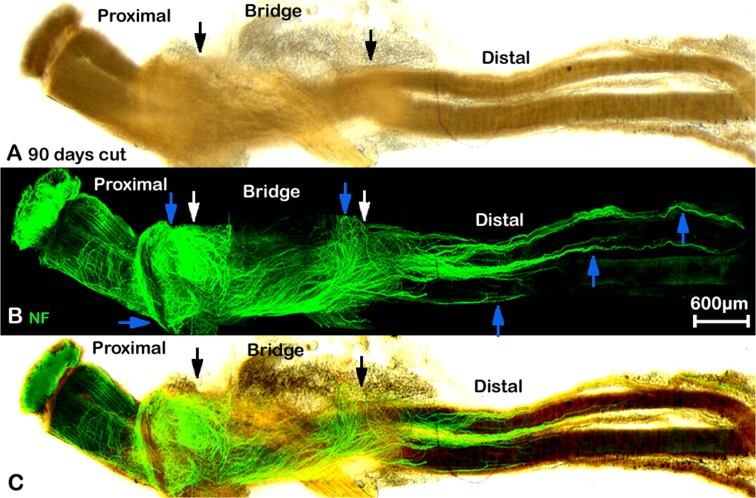
Whole mount staining of transected sciatic nerve preparation after 90 days injury showing misdirected regrowing axons. A: phase image showing the structure of transected sciatic nerve, black arrows mark the proximal (left) and distal (right) nerve stumps. B: neurofilament (NF) antibody whole mount staining show regenerating axons in the nerve bridge and misdirected regrowing axons in both proximal and distal nerve stumps, blue arrows indicate that axons are growing outside of both proximal and distal nerve stumps. White arrows mark the proximal (left) and distal (right) nerve stumps. C: merged image of panels A and B.

Comparison of the diameter of the singly migrating axons (2.122±0.335μm) with the diameter of large bundles at day 7 (7.651±0.787μm) revealed a significant difference (P<0.0001, n = 30 from 5 different animals). Within the possible inaccuracies of these measurements, it would still suggest that the population of axons crossing the nerve bridge is different to those axon bundles growing perpendicular to the bridge, although staining with markers of sensory and motor neurons clearly needs to be done if any functional significance is to be attributed to this phenomenon. By 10 days after transection, a number of the large axon bundles are still present and appear to have turned now through 180 degrees and are growing back over the proximal stump (Fig. 4B, blue arrows). As described above, in these preparations, the intact epineurium prevents antibody penetration and staining of axons inside the proximal stump of the nerve or those regrowing axons inside the distal stump of the nerve. This means that neurofilament positive axons seen at 10 days after transection are actually growing outside of the nerve trunk (Fig. 4B). Thus, there are two populations of misguided axons visualised here, one smaller population turns back and grows along the outside of the proximal nerve segment, and another larger population, that has managed to cross the nerve bridge but fails to enter the distal stump and instead grows along the outside of distal nerve segment. Both populations can also be seen clearly at 14 days and three months after nerve transection (Figs. 5B and 7B, blue arrows).

### Visualizing the Schwann cell-axon partnership in regenerating nerves

Using whole mount staining, we can clearly see in great detail the interactions between Schwann cells and axons in the regenerating nerve. Four days after nerve transection, when there is no apparent axonal growth, the growth cones of the axonal front were covered by Schwann cell processes (Fig. 6A). On day 5, only axons forming large bundles are growing into the nerve bridge and the leading edge of axonal bundles were covered by Schwann cell processes often forming a ball shape (Figs. 3A, 6B and 6E). At day 5 (Fig. 3A), significant numbers of S100β positive Schwann cells can be seen migrating from the distal nerve stump into the bridge and by day 7 these distal Schwann cells can be seen interacting with axons that have crossed the nerve bridge (Fig. 3C and 3D). Some Schwann cells are aligned completely with axons (Fig. 3C and 3D). Before regenerating axons start to interact with distal migrating Schwann cells, we have observed that proximal Schwann cell leading processes appear to proceed in front of the re-growing neurofilament-positive axons, indicating that proximal Schwann cell leading processes are required for guiding axons across the nerve bridge. Thus, our findings confirm the previous data [9,12] showing that it is Schwann cells that lead the way during regeneration and axons appear to follow after transection injury. In the nerve bridge at 10d after transection, Schwann cell leading processes proceed in front of axons, while the Schwann cell bodies, as visualized by Hoechst staining, are held by axons crossing the nerve bridge (Fig. 6D and 6H).

### Angiogenesis following nerve transection

**Fig 8:**
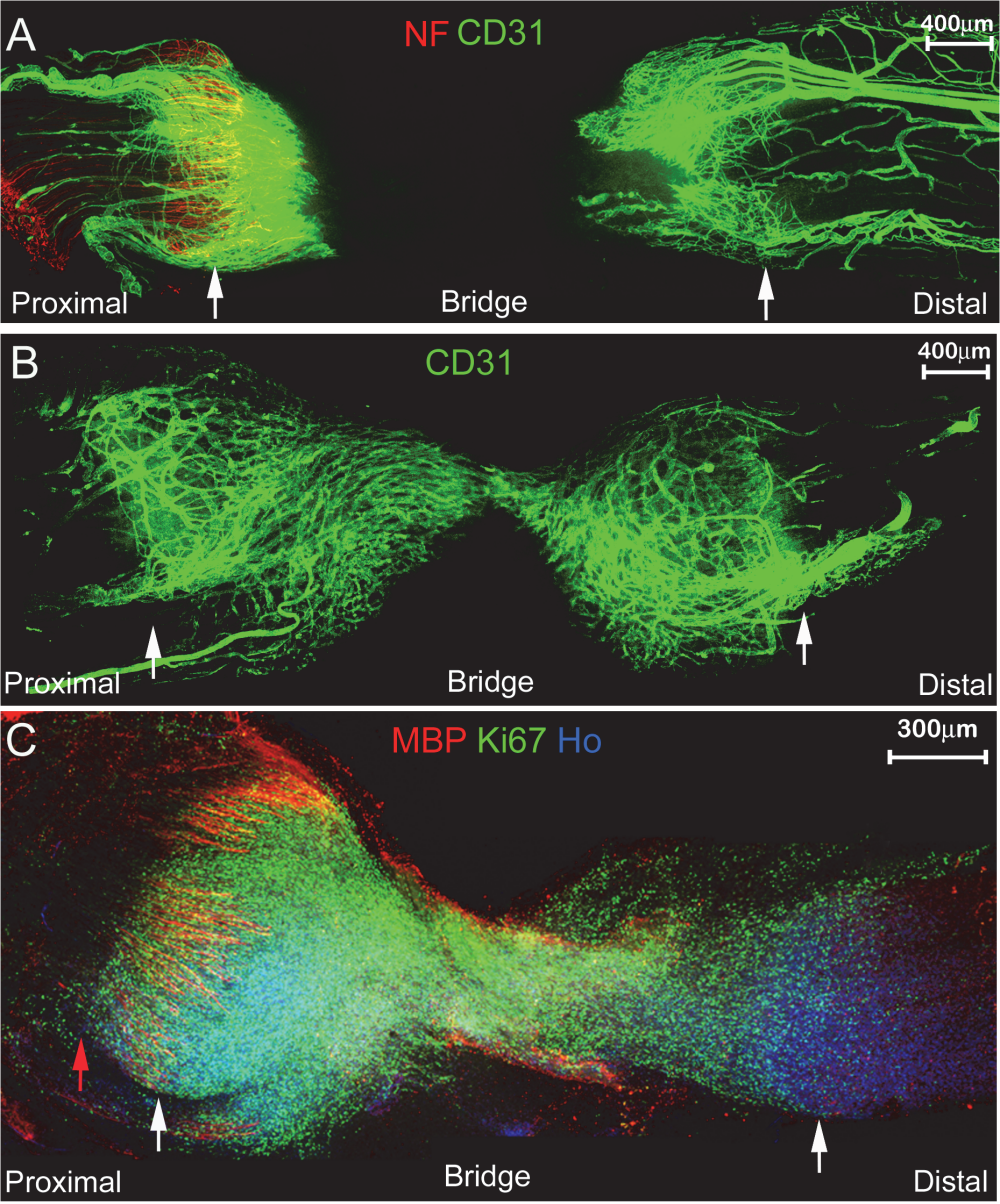
Whole mount staining showing angiogenesis and cell proliferation following nerve transection. A and B: staining with the endothelial cell marker CD31 and neurofilament (NF) at 5 days (A) or 6 days (B) after nerve transection identifies new blood vessel formation at the proximal and distal stumps of the transected nerve (identified by white arrows). C: whole mount sciatic nerve staining with myelin basic protein (MBP) and Ki67 antibodies to show presence of myelinated Schwann cells in proximal stump (MBP) and cell proliferation (Ki67) at 7 days after transection injury. Nuclei are counterstained with Hoechst dye (Ho). White arrows mark the proximal (left) and distal (right) nerve stumps.

Following nerve transection injuries, there is an increase in vascular endothelial growth factor A in the recruited macrophages and neutrophils within the nerve, which stimulates angiogenesis within the damaged nerve [14, 15]. Using the endothelial cell marker CD31, we have examined blood vessel formation after nerve transection by whole mount staining at 5 and 6 day timepoints. At 5 days after transection, blood vessel formation can clearly be seen in both proximal and distal stumps of the nerve (Fig. 8A). At 6 days, the network of CD31 positive blood vessels between the two nerve stumps has linked up (Fig. 8B).

To further demonstrate the use of the whole mount staining protocol following nerve transection, we have also stained nerve preparations using Ki67 and myelin basic protein (MBP) to detect cell proliferation and myelinated Schwann cells respectively within the regenerating nerve at 7 days after transection. Fig. 8C shows the huge numbers of proliferating Ki67 positive cells within the nerve and nerve bridge and the presence of intact MBP positive myelin sheaths in the proximal stump of the transected nerve.

## Discussion

The potential for the peripheral nervous system to repair is impressive but in practice, particularly after nerve cut, the amount of functional repair is often very poor. The ability to understand the relationship between regenerating axons, Schwann cells, nerve fibroblasts, together with events driving the formation of the nerve bridge is key to understanding and boosting these processes to improve upon the poor outcomes frequently seen in patients with peripheral nerve injuries [9, 16]. Using a relatively simple modification of a whole mount staining protocol, we have shown how this can be applied to the study of axonal regeneration, cell proliferation, Schwann cell-axon interaction and angiogenesis in the peripheral nerve following injury. This, we hope, will allow a much more detailed analysis of the events occurring during this process than was previously possible. A number of different protocols have been published that allow 3D imaging of tissue samples, for instance the *Scale* clearing agent [17] or the 3D Imaging of Solvent-Cleared Organs (3DISCO) [18]. In the case of 3DISCO, the use of the final clearing agent sharply reduced the half-life of the green fluorescent protein (GFP) in cells. The simple glycerol clearing protocol we have used for this work is as effective as the *Scale* urea-based reagent in our hands and is very effective for the relatively smaller tissue samples. We have use neurofilament staining to measure axonal regeneration in our experiments, but sensory neuron-specific markers, for example advillin [19], could be used to determine whether there are differences in the regeneration of sensory neurons in these nerve injury paradigms. A growing number of mouse mutants show defects in PNS regeneration following injury [8, 20–25], and the future application of such a whole mount staining protocol to such transgenic mouse models will, we hope, be useful in understanding the processes of peripheral nerve regeneration and functional repair. The whole mount protocol in this paper has recently been used to measure changes in axonal regeneration underlying the age-related decline in PNS repair [26]. A number of studies of PNS repair have used the approach of using transgenic animals expressing neuron specific fluorescent proteins, driven by the Thy-1 promoter, for example driving yellow fluorescent protein (YFP) in a subset of neurons alone [10, 27] or driving cyan fluoresecent protein (CFP) in neurons in combination with Schwann cell-specific expression of green fluoresecent protein (GFP) [28]. Such studies do have the advantage that they may not require antibody labelling of samples, but will require several extra steps in mouse breeding to allow their use for the analysis of repair in knockout mice with Schwann cell or neuron-specific deletions of genes. Alternatively, the combined use of such transgenics expressing neuronal YFP or CFP in combination with our whole mount protocol may be particularly useful for staining multiple cell types or markers within a single preparation.

A previous study has reported a very close relationship between proximal stump outgrowth of axons and Schwann cells. In particular, using the Schwann cell marker, glial fibrillary acidic protein (GFAP), long Schwann cell processes have been observed proceeding in front of the regenerating axons [17]. Using the whole mount staining protocol, we have been able to confirm this phenomenon more clearly at various time points following injury. The ability of Schwann cells in the proximal stump to extend long leading processes and align with axons would appear to be crucial for axons to regrow across the nerve bridge following a transection injury.

Without microsurgical repair, a transection injury to a peripheral nerve is likely to lead to a permanent loss of both motor and sensory function. Possible reasons for this have been explained as neuronal death following an injury and as well as the misdirection of regenerating axons within the distal nerve to inappropriate targets [9, 10, 29]. Using the whole mount staining technique, we have now observed an extensive axon guidance defect around the nerve bridge with a large population of axons growing along outside of both the proximal the distal nerve trunks. This new finding identifies yet another potential cause of the failure of nerve repair following transection injuries.

In summary, the whole mount staining protocol we describe will, we hope, provide a novel and more accurate way to measure the processes of axonal regeneration, Schwann cell-axon interaction and angiogenesis within the repairing peripheral nerve.
